# Lipid accumulation product (LAP) index for the diagnosis of nonalcoholic fatty liver disease (NAFLD): a systematic review and meta-analysis

**DOI:** 10.1186/s12944-023-01802-6

**Published:** 2023-03-15

**Authors:** Menooa Ebrahimi, Seyed Arsalan Seyedi, Seyed Ali Nabipoorashrafi, Soghra Rabizadeh, Mojdeh Sarzaeim, Amirhossein Yadegar, Fatemeh Mohammadi, Razman Arabzadeh Bahri, Peyman Pakravan, Paria Shafiekhani, Manouchehr Nakhjavani, Alireza Esteghamati

**Affiliations:** 1 Endocrinology and Metabolism Research Center (EMRC), School of Medicine, Vali-Asr Hospital, Tehran, P.O. Box 13145784 Iran; 2 Research Center for Immunodeficiencies, Children’s Medical Center, Tehran University of Medical Sciences, Tehran, Iran

**Keywords:** Insulin resistance, Lipid accumulation product (LAP), Liver disease (NAFLD), Meta-analysis, Nonalcoholic fatty liver disease

## Abstract

**Background:**

Lipid accumulation product (LAP) is an index calculated by waist circumference (WC) and triglyceride (TG), which reflects lipid toxicity. This study aims to investigate the association between the LAP index and nonalcoholic fatty liver disease (NAFLD) in a systematic review and meta-analysis.

**Methods and results:**

PubMed, Scopus, and Web of Science online databases were searched for eligible studies that investigated the association of the LAP index and NAFLD. Sixteen observational studies with 96,101 participants, including four cohort studies, one case‒control study and 11 cross-sectional studies with baseline data, were entered into this analysis. Fourteen studies reported a significant association between the LAP index and NAFLD, and two reported that this relation was not significant; two different meta-analyses (1- mean difference (MD) and 2- bivariate diagnostic test accuracy [DTA]) were conducted using Stata version 14. The LAP index was compared in subjects with and without NAFLD, and the difference was significant with 34.90 units (CI 95: 30.59–39.31, *P* < 0.001) of the LAP index. The DTA meta-analysis was conducted and showed that the LAP index pooled sensitivity and specificity for screening of NAFLD were 94% (CI95: 72%–99%, I^2^ = 99%, *P* < 0.001) and 85% (CI95: 62%–96%, I^2^ = 99%, *P* < 0.001), respectively.

**Conclusion:**

The LAP Index is an inexpensive, sensitive, and specific method to evaluate NAFLD and may be valuable for NAFLD screening.

**Supplementary Information:**

The online version contains supplementary material available at 10.1186/s12944-023-01802-6.

## Introduction

NAFLD is the leading cause of chronic liver disease around the globe [1]. NAFLD affects 25–32% of the population (5–18% in Asia and 20–30% in Western countries) [2]. NAFLD/nonalcoholic steatohepatitis (NASH) incidence grew by 1.35% annually, from 19.34 million in 1990 to 29.49 million in 2017 worldwide [3]. Risk factors for NAFLD are obesity, dyslipidemia, insulin resistance (IR), and hypertension [4, 5].

NAFLD is defined as fat accumulation in 5% of hepatocytes or more, without daily alcohol consumption of greater than 20 g for females and 30 g for males or other causes of fatty liver [6]. NAFLD histologically ranges from simple steatosis to NASH (hallmarks are fatty changes, inflammation, and ballooning of hepatocytes), advanced fibrosis, cirrhosis, and hepatic failure, which can ultimately lead to hepatocellular carcinoma (HCC) [7–11].

Biopsy of the liver is the method of choice for diagnosing NAFLD and liver cirrhosis. However, this method is invasive and is not a suitable tool for follow-up [12]. Other diagnostic methods for NAFLD are the NAFLD liver fat score (with 86% sensitivity and 71% specificity), ultrasonography, which is less accurate in cases of mild steatosis, and magnetic resonance imaging proton density fat fraction (MRI-PDFF), which is more precise but more expensive and only available in limited quantities [12–15].

Treatment of NAFLD is mainly based on lifestyle modifications such as weight loss, a low-calorie diet, and aerobic exercise; these modifications lower hepatic fat accumulation and improve liver metabolism [16–19]. There are no approved medications for NAFLD, and in the late stages and cirrhosis, a liver transplant is the only treatment [20, 21].

LAP is an index for estimation of excessive lipid accumulation, which is calculated by WC and fasting plasma TG: (LAP = (WC (cm) – 65) x TG (mmol/L)) for men, and (LAP = (WC (cm) – 58) x TG (mmol/L)) for women [22]. Recently, some articles have shown that LAP may be an indicator of type 2 diabetes (T2D), IR, metabolic syndrome (MetS), and NAFLD in the general population and may be related to the risk of cardiovascular events [22–25]. However, some other studies did not agree with this association regarding NAFLD [26, 27].

As a low-cost and safe method, the LAP index might be an option for detecting NAFLD compared to invasive and expensive procedures such as liver biopsy and MRI-PDFF. Based on our search, no systematic review regarding the relationship between NAFLD and the LAP index has been performed previously. This systematic review aimed to investigate the articles and assess whether the LAP index is reliable for NAFLD screening.

## Methods

The Preferred Reporting Items for Systematic Reviews and Meta-Analyses (PRISMA) statement 2020 guidelines were used to conduct this systematic review [28].

This systematic review protocol was registered in the International Prospective Register of Systematic Reviews (PROSPERO); CRD42022334204.

### Search

Three online databases, including Scopus, Web of Science, and PubMed, were searched systematically until September 2022. Search strings were relevant to the LAP index and NAFLD (Supplementary file).

This systematic search was performed without any language limitations.

### Study selection

#### Eligibility criteria

We included human studies based on the following PICO. (a) Population: Adult participants (≥ 18 years old) with NAFLD. (b) Intervention: LAP index. (c) Control: Adult participants without any types of NAFLD. (d) Outcomes: The prognostic performance of the LAP index for the diagnosis of NAFLD.

This study included original, observational, and peer-reviewed papers with the mentioned PICO. Additionally, studies reporting at least the means of the LAP index among subjects with and without NAFLD were eligible. Additionally, articles detecting NAFLD only by a common diagnostic method (e.g., CT scan, ultrasonography, biopsy, Chinese diagnostic criteria) were included.

#### Exclusion criteria

In this study, articles with inadequate information, such as sample size (total participants with and without NAFLD) or standard deviation (SD), were not eligible for this study. In addition, studies with a specific disease or condition as inclusion criteria (e.g., obesity, polycystic ovary, T2D) were not eligible for this meta-analysis since it was performed on the general population. Additionally, review articles, conferences, preprint papers, abstracts, dissertations, reports, randomized control trials, editorials letters, and chapters were not included.

Based on the aforementioned criteria, two independent authors (M. S and M. E) screened the title/abstract, and then, for the remaining records, full texts were reviewed. Regarding the dispute, the two reviewers discussed reaching an agreement. If they could not reach an agreement, the third reviewer resolved disagreements (SA. N).

### Data extraction

Two independent reviewers extracted data (M. E and R. AB) from each included publication. If they could not reach an agreement regarding an item, the third reviewer resolved disagreements (SA. S).

The following data were extracted: the first author’s name of the publication, publication year, design of the study, location of study, important exclusion and inclusion criteria for each study, total case and control number, LAP index mean, and SD of case and control groups, the female to male ratio, and the range age and mean age of subjects. Additionally, the specificity and sensitivity of the LAP index for diagnosing NAFLD were written in the predesigned Google worksheet. In addition, if the LAP index was reported in mg/dl units, it was converted to mmol/l for meta-analysis.

### Quality assessment

The Newcastle–Ottawa Scale (NOS) was used to evaluate the quality of the included studies. This tool evaluates three main elements, including 1- sample selection, 2- comparability of the sample based on the analysis or design, and 3- how the exposure was defined or how outcomes of interest were diagnosed [29]. Studies can achieve one star for each numbered item regarding selection and exposure domains maximally. The comparability domain receives two stars at maximum. High quality was considered as achieving six stars or more.

### Statistical analysis

Statistical software for data science version 14 through the "Midas", "Metandi" and "Metan" commands were utilized for DTA and MD meta-analysis. The analysis was performed via a random-effects model (a model considered that the true effect might be different from one study to another due to the heterogeneity and differences among studies). Studies that computed the LAP index for the diagnosis of NAFLD were recorded in the MD meta-analysis. If the SDs were not declared in a study, the interquartile range (IQR) or 95% confidence interval (CI) was used to compute the SD using the formulas listed below [30]:

$$1-\;\mathrm{Using}\;\mathrm{IQR}:\;\mathrm{SD}\;=\;\left(\mathrm q3\;-\;\mathrm q1\right)/1.35,\;\mathrm{and}\;2-\;\mathrm{Using}\;\mathrm{CI}:\:\mathrm{SE}\;=\;\mathrm{SD}\;=\;\left(\left[\mathrm{CI}\;\mathrm{upper}\;\mathrm{limit}\right]\;-\;\left[\mathrm{CI}\;\mathrm{lower}\;\mathrm{limit}\right]\right)\ast\sqrt{\mathrm N/3.92}$$

Studies that calculated the specificity and sensitivity of the LAP index for diagnosing NAFLD were selected for DTA meta-analysis. Bivariate DTA meta-analysis was conducted to calculate pooled sensitivity and specificity; this model is applied when different cutoffs are reported in studies. The Cochran-Q test and I^2^ index were applied to assess the heterogeneity of the meta-analysis. Significant heterogeneity of data was defined as I^2^ > 50% or a significant Cochran-Q test (*P* < 0.10). To evaluate the effects of possible confounding factors on the heterogeneity among publications, subgroup analyses were performed. NAFLD diagnostic method and geographic area (country and continent) of studies were used for subgroup analysis as categorical variables. Additionally, meta-regressions on continuous variables (studies’ mean age of subjects, female-to-male proportion, and publication year) were performed.

### Publication bias

Publication bias was assessed through funnel plots and Begg’s, Egger’s and Deek’s tests with significance levels at *P* < 0.05 [31, 32].

## Results

### Study characteristics

**Fig. 1:**
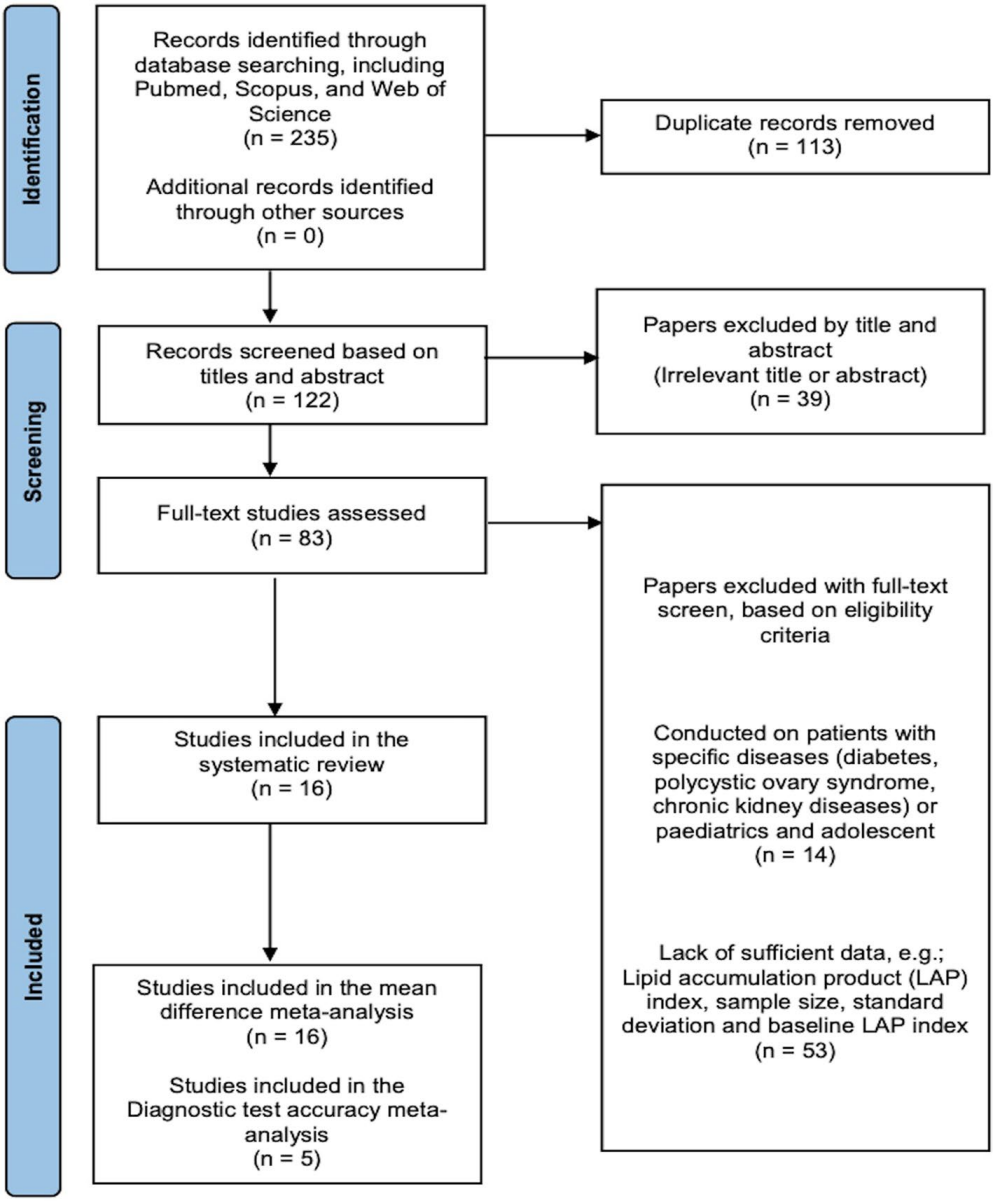
PRISMA chart

After the primary search and eliminating duplicate records, 122 unique results were identified. In the next step, 39 studies were excluded by screening titles or abstracts. Throughout screening by titles/abstracts, reviewers had an extremely conservative approach. Finally, 83 remaining studies underwent a full-text evaluation. Based on the remaining articles' relevance to this study's purpose and the predefined eligibility criteria for this review, 16 studies were selected for this study (Fig. 1) [22, 26, 27, 33–45].

Of the total participants with 96,101, 30,665 had NAFLD, and 65,436 did not. The studies were conducted in different geographic areas. Regarding the design of studies, most studies were cross-sectional [22, 27, 33, 35–38, 40, 41, 43, 45]. Additionally, four cohort [34, 39, 42, 44] and one case‒control [26] study were selected for this study, as they report baseline tables, and their data could be added to this study [26, 34, 39, 42, 44].

The mean participants’ age for the selected study ranged between 29.8 and 76.3 years of age, and their sample size varied from 55 to 40,459 participants.

Different methods were used to diagnose NAFLD in studies; the most common one was ultrasonography (*N* = 15), and only one study used Chinese diagnostic criteria to detect patients with NAFLD [37]. The Chinese diagnostic criteria employ two major components for the diagnosis of NAFLD: (i) liver imaging study meeting diffuse fatty liver criteria and not explained by any other causes; (ii) individuals with components of metabolic syndrome with continuous elevation of ALT or AST and GGT or both from an unknown cause for more than 6 months; and finally, if abnormal fatty liver imaging or zymogram shows improvement after weight reduction and improvement of insulin resistance occurs, the diagnosis of NAFLD is definite. All these criteria are applicable only in the absence of any other disease that can lead to liver steatosis [46].

In this study, we performed two different meta-analyses. 1- MD meta-analysis to evaluate whether the LAP index is different between participants with and without NAFLD, 2- DTA meta-analysis to evaluate the screening precision of the LAP index for NAFLD.

### Mean difference

**Fig. 2:**
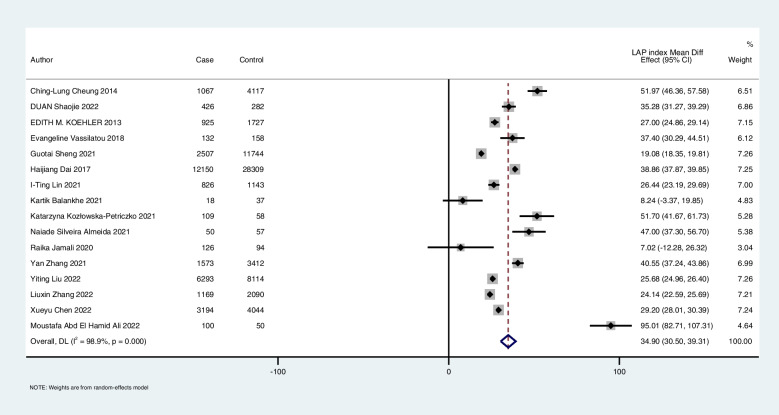
The pooled mean difference in the LAP index between participants with and without NAFLD (forest plot)

The MD meta-analysis (random-effect model) of 16 selected articles demonstrated that the pooled mean LAP index in subjects with NAFLD was 34.90 units (CI 95: 30.59–39.31) higher than that in participants without NAFLD (Fig. 2). The result of this analysis remained significant when leave-one-out was performed. The influence test evaluates whether only one study has a significant impact on the total result. The results of I2 (98.9%) and the Cochran-Q test (*P* < 0.001) showed high heterogeneity in this meta-analysis.

Subgroup analyses were conducted based on the NAFLD diagnostic method and geographic area of studies (Table 2). Additionally, meta-regressions on the studies’ mean age of subjects, publication year, and female-to-male proportion did not reveal any important association.

**Fig. 3:**
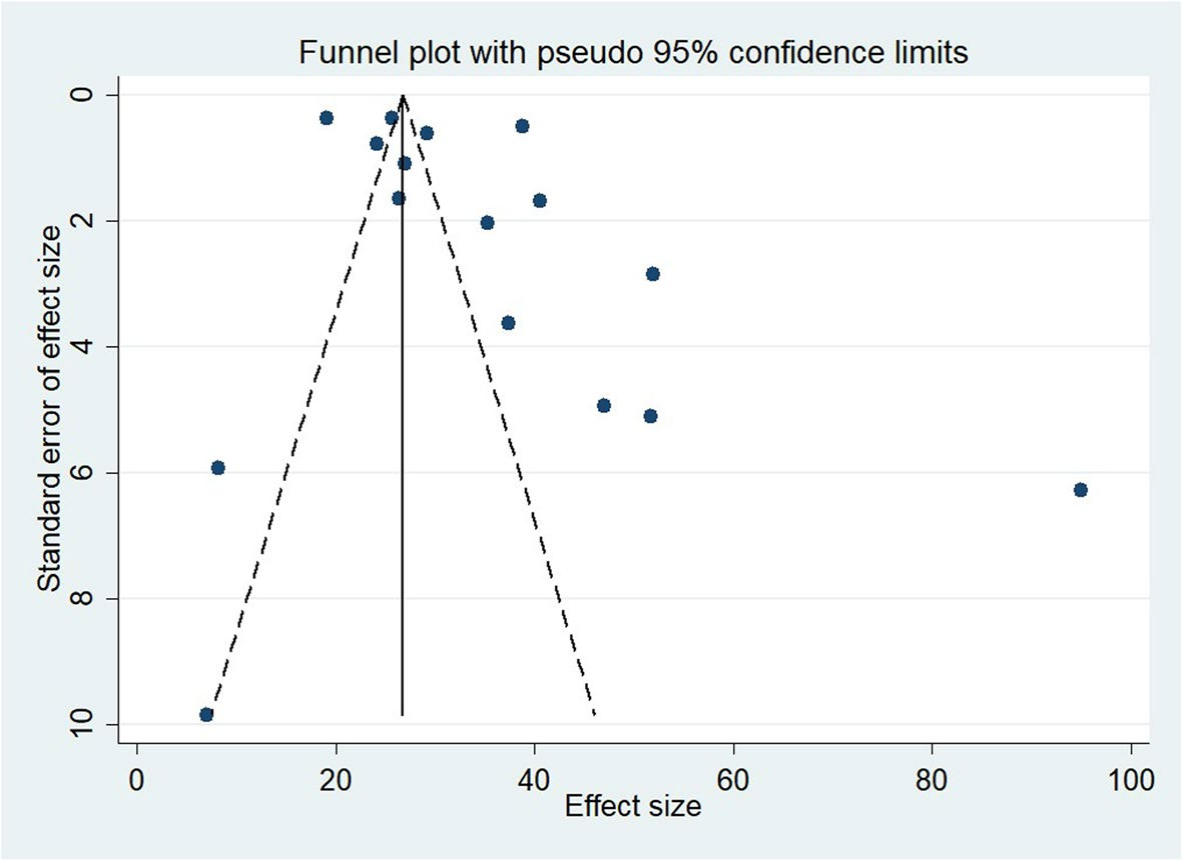
Funnel plots assessing publication bias among the included studies

Begg’s and Egger’s regression tests (Supplementary file) and visual observation of the funnel plots did not show a significant effect of publication bias on the MD meta-analysis (Fig. 3).

### DTA of LAP index

Five studies with 17,934 participants were selected in the DTA meta-analysis [22, 33, 35, 38, 39]. Variant cutoffs for the LAP index were defined in various articles; however, there was no significant association between sensitivity and specificity among reported data between different studies (*P* = 0.624). Therefore, a bivariate DTA meta-analysis was conducted to evaluate the combined specificity and sensitivity of the LAP index in screening participants for NAFLD.

**Fig. 4:**
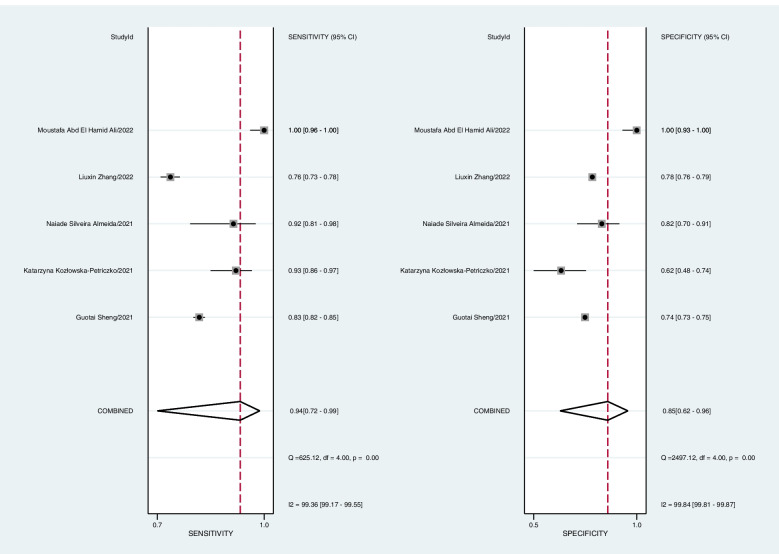
The combined specificity and sensitivity of the LAP index for NAFLD screening (forest plots)

Pooled sensitivity at 94% (CI95: 72%—99%, I 2 = 99%, *P* < 0.001) and specificity at 85% (CI95: 62%—96%, I 2 = 99%, *P* < 0.001) were calculated. (Fig. 4).

**Fig. 5:**
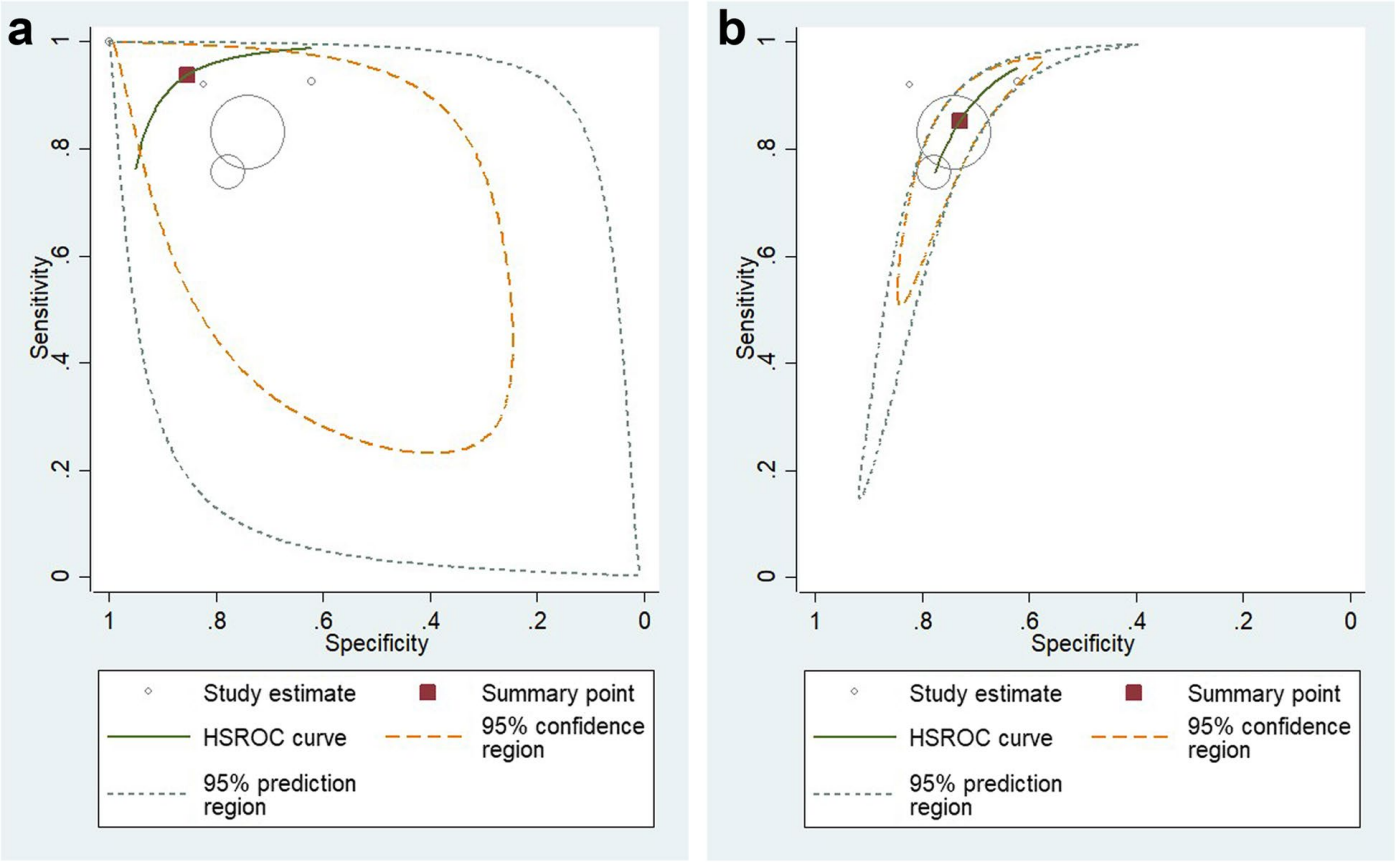
sROC curve for the accuracy of the LAP index in studies (**a**) reported data totally (**b**) reported data without Moustafa Abd El Hamid Ali [33]

Additionally, the Summary Receiver Operating Characteristic (sROC) curve was plotted, and the Area Under the Curve (AUC) was 0.95 (CI 95: 0.93—0.97), which showed that the LAP index is accurate for the screening of NAFLD (Fig. 5).

The heterogeneity of the sROC curve is defined by visual inspection. Observation of the area between the confidence and prediction regions showed that the heterogeneity of the sROC curve is moderate to high [47]. Based on the sROC graph, one of the studies that reported both specificity and sensitivity of the LAP index for diagnosis of NAFLD 100% [33] might be the main source of this heterogeneity, so another analysis with remaining included studies was performed. The second DTA meta-analysis with four studies revealed 85% (CI95: 77%—91%, I 2 = 69%, *P* < 0.001) sensitivity and 73% (CI95: 68%—77%, I 2 = 27%, *P* = 0.16) specificity of the LAP index for the screening of NAFLD, and the heterogeneity was reduced significantly based on HSROC. The new sROC curve was graphed with an AUC of 0.82 (CI 95: 0.79—0.85). Based on the pooled odds ratio, the population with a higher LAP index was at 15.52 (10.96 – 21.97) times more risk of NAFLD.

Deeks’ funnel plot did not reveal any substantial publication bias regarding the DTA meta-analysis (*P* = 0.25).

### Methodological quality

Except for two studies [27, 33], which achieved five stars, study quality ranged from 6 to 8 stars, and based on NOS, they were considered good to high-quality studies (Table 1). In general, publications received the most stars in the domain of exposure and the fewest stars in the domain of selection. Additionally, three studies did not receive any stars in the comparability domain. (Supplementary file; Table 1).

**Table 1:** Characteristics of the included studies

Author, year	Country	Study design	Total case (Female to male ratio)	LAP index in cases; Mean (SD)	Total control	LAP index in control; Mean (SD)	Important inclusion/exclusion criteria	NAFLDdiagnostic method	Sensitivity	Specificity	Cutoff value	NOSscore	Age range (Age mean)
Ching-Lung Cheung [44], 2014	America	LS	1067 (46/54)	91.71 (89.24)	4117	39.74 (54.34)	NR	Ultrasonography	NR	NR	NR	8	20 to 74 (41.42)
DUAN Shaojie [43], 2022	China	CS	426 (24/76)	61.3 (35.29)	282	26.02 (18.88)	NR	Ultrasonography	Men: 63.7 Women: 79.8	Men: 81.1 Women: 85.0	Men: 50.88 Women: 50.91	6	≥ 18 (39.16)
EDITH M. KOEHLER [42], 2013	Netherlands	LS	925 (61/39)	58 (30.37)	1727	31 (18.52)	conducted among elderly inhabitants	Ultrasonography	NR	NR	NR	7	NR (76.3)
Evangeline Vassilatou [41], 2018	Greece	CS	132 (100/0-Only female)	61.1 (39.3)	158	23.7 (15.2)	premenopausal women aged 18–45 years	Ultrasonography	NR	NR	NR	6	18–45 (29.8)
Guotai Sheng [22], 2021	Japan	CS	2507 (19/81)	26.96 (18.24)	11,744	7.88 (7.56)	NR	Ultrasonography	83.13	74.04	NR	6	≥ 18 (43.53)
Haijiang Dai [45], 2017	China	CS	12,150 (34/66)	58.03 (54.19)	28,309	19.17 (18.12)	NR	Ultrasonography	Men: 77% Women: 82%	Men: 75% Women: 79%	Men: 30.5 Women: 23	6	≥ 18 (43.72)
I-Ting Lin [40], 2021	Taiwan	CS	826 (57/43)	51.06 (43.62)	1143	24.62 (22.78)	NR	Ultrasonography	NR	NR	NR	6	NR (54.9)
Kartik Balankhe [27], 2021	India	CS	18 (NR)	53.67 (21.26)	37	45.43 (19.2)	nonobese (BMI < 25 kg/1.76 m2), less than sixty years of age individuals	Ultrasonography	NR	NR	NR	5	< 60 (43.4)
Katarzyna Kozłowska-Petriczko [39], 2021	Poland	LS	109 (61/39)	76.3 (49.2)	58	24.6 (15.2)	NR	Ultrasonography	93	62	23 (Female) 30.5 (Male)	8	≥ 18 (52.6)
Naiade Silveira Almeida [38], 2021	Brazil	CS	50 (68/32)	61.10 (33.40)	57	14.1 (11.11)	NR	Ultrasonography	91	82	26.7	6	18–60 (39.71)
Raika Jamali [26], 2020	Iran	LS	126 (46/54)	110.67 (80.05)	94	103.65 (65.81)	NR	Ultrasonography	NR	NR	NR	8	NR (51.6)
Yan Zhang [37], 2021	China	CS	1573 (68/32)	79.91 (61.7205)	3412	39.36 (38.2212)	Study screened subjects over 65 years	Chinese Diagnostic Criteria	71(F) 79.8(M)	67(F) 70.4 (M)	49.17(Female) 36.15 (Male)	6	66–115 (73.8)
Yiting Liu [36], 2022	China	CS	6293 (32/68)	38.25 (27.57)	8114	12.57 (10.83)	NR	Ultrasonography	82(F) 69 (M)	74(F) 79 (M)	19.2(Female) 27.86 (Male)	6	NR (47)
Liuxin Zhang [35], 2022	China	CS	1169 (14/86)	40.59 (25.55)	2090	16.45 (11.71)	NR	Ultrasonography	75.6 84.4(F) 77 (M)	77.7 77.9(F) 73 (M)	26.9 19.7(Female) 26.9 (Male)	7	NR (40.59)
Xueyu Chen [34], 2022	China	LS	3194 (39/61)	47.0 (32.0)	4044	17.9 (13.64)	NR	Ultrasonography	NR	NR	NR	7	NR (41.6)
Moustafa Abd El Hamid Ali [33], 2022	Egypt	CS	100 (59/41)	113.77 (61.90)	50	18.76 (7.23)	NR	Ultrasonography	100	100	33.2	5	18–75 (42.72)

*NOS* Newcastle‒Ottawa Scale, *LS* longitudinal study, *CS* cross-sectional study, *NR* not reported

## Discussion

This study is the first meta-analysis to provide evidence of the relationship between the LAP index, a useful formula for estimating body fat accumulation, and NAFLD. Fourteen of the included studies reported a significant association between the LAP index and NAFLD; however, two studies reported that this association was not significant. Although the MD of the LAP index was controversial among studies, this meta-analysis showed that the mean LAP index was considerably higher in individuals with NAFLD. The results of the MD meta-analysis showed a significant difference in the LAP index between those with and without NAFLD (*P* < 0.001); however, high heterogeneity was observed in the results. Therefore, we performed subgroup and meta-regression analyses. Subgroup analysis based on geographical areas (continent where the study was performed) reduced heterogeneity (Table 2). However, some potential factors might cause heterogeneity that could not be evaluated through subgroup analysis, including duration and stage of NAFLD, smoking, and underlying diseases in participants.

**Table 2:** Subgroup meta-analysis by geographic area and NAFLD diagnostic method

Subgroup analysis	N	Pooled mean difference (95% CI)	I^2^ (*P*)
Geographic area			
America	2	50.73 (45.87–55.58)	0 (0.385)
Asia	11	31.68 (26.35–36.59)	99.2 (*P* < 0.001)
Europe	3	37.97 (26.45–36.90)	92.9 (*P* < 0.001)
Country			
China	6	32.18(26.56–37.81)	99.1 (*P* < 0.001)
NAFLD diagnostic criteria			
Ultrasonography	15	34.46 (29.95–38.97)	98.9% (*P* < 0.001)

Recently, a large number of studies have been conducted on NAFLD due to its increasing prevalence; reports indicate a prevalence of 27 to 34% in North America, 8 to 45% in various European nations, and 15 to 38% in Asia [48–55]. The rising prevalence of NAFLD is related to the increased incidence of a sedentary lifestyle, obesity, dyslipidemia, T2D, and MetS [56–58]. NAFLD is a health burden associated with obesity, T2D, and MetS, and it is proposed that patients with similar difficulties should be screened for NAFLD [58, 59].

NAFLD is a chronic disease that takes years to develop. Thus, regular screening and well-timed diagnosis of NAFLD in young people can prevent major complications, including hepatocellular carcinoma and cirrhosis [45]. As mentioned earlier, currently, the diagnosis and screening of NAFLD are high-cost and not available everywhere; therefore, we need a simple method with a lower cost for the general population [23, 60].

Two of the major indicators of NAFLD are abdominal obesity and serum TG levels [61, 62]. In abdominal obesity, adipocytes generate numerous adipokines and cytokines, including leptin, adiponectin, resistin, visfatin, and chemerin [63]. High concentrations of resistin and leptin and low concentrations of adiponectin are associated with insulin resistance [64]. Excess adipocytes contribute to a chronic inflammatory response by activating the proinflammatory signaling pathway and abnormal cytokine production; finally, these pathophysiological changes may cause progress toward NAFLD development [65]. Recently, several studies have shown that probiotics and omega-3 can improve liver enzymes and clinical and metabolic markers in patients with NAFLD [66–69]. Additionally, it has been demonstrated that omega-3 fatty acids can reduce liver steatosis by reducing TG levels; this fact supports the role of elevated TG in the development of NAFLD [70].

The LAP index, a marker for the evaluation of excess lipid accumulation and a clinically useful marker for the estimation of insulin resistance, was first presented by Kahn [23, 71]. Over recent years, multiple studies have discovered a significant correlation between the LAP index and cardiometabolic risk factors [60, 72]. In a cross-sectional study, Taverna et al. [73] revealed that the LAP index has high accuracy in the diagnosis of MetS, and as shown in previous studies, MetS is also associated with NAFLD [73, 74]. Furthermore, Xia et al. found that LAP is a suitable marker for diagnosing insulin resistance in nondiabetic patients [25]. Shi et al. also demonstrated a positive association between the LAP index and arterial stiffness [75]. Similarly, patients with NAFLD have increased arterial stiffness [76].

Accordingly, it is reasonable that the LAP index, which is based on WC and TG, is considerably related to NAFLD. In recent years, several studies have mentioned the LAP index as a suitable indicator for the diagnosis of Mets and NAFLD in adults, which gives rise to the importance of conducting a systematic review and meta-analysis on this issue [45, 73, 77].

Previous studies on the LAP index and NAFLD and the MD meta-analysis that was performed. in this study suggested that LAP could be a reliable marker for the screening of NAFLD.

Therefore, a meta-analysis on DTA was performed. Studies that reported sensitivity. and specificity (or data that these variables can be extracted through) were selected for the DTA meta-analysis to calculate the pooled specificity, sensitivity, and AUC of the LAP index for diagnosing NAFLD. This analysis showed that despite the straightforward calculation of the LAP index, the high specificity and sensitivity of this index for diagnosing NAFLD and candidates it a valuable tool for its screening in populations.

### Strengths and limitations

This study has some strengths. First, it has extensive and replicable methods for searching published literature. Second, it is the first systematic review and meta-analysis on the LAP index and NALFD in the literature. Third, by including 96,101 individuals with and without NAFLD, a conclusive result with high precision and low bias may have been achieved for the general population in this study. Finally, in addition to resolving the conflict between the existing studies on the relationship between the LAP index and NAFLD, this article introduces the LAP index as an appropriate and low-cost method for NAFLD screening with high sensitivity and specificity.

There are some limitations to this study, similar to other systematic reviews. Laboratory data reported in each study were measured in different lab centers with various facilities, and different alcohol consumption limits were defined for each specific study, which may lead to some inconsistency. In addition, suggesting different cutoffs by each study could cause some discrepancies in the final result. Additionally, ultrasonography, an operator-dependent method for diagnosing NAFLD, could result in disparities in interpretation and some cases being missed [78]. Some other disadvantages of the meta-analysis are that it integrates various kinds of research, and the overall effect can overlook significant variations. Although the random-effect model was used for MD meta-analysis to address high heterogeneity, it could not eliminate this issue completely. Finally, the publication bias test did not show any relevant bias; however, the lack of unpublished studies could have led to publication bias.

## Conclusions

These data support the use of the LAP index for the diagnosis of NAFLD in the general population as an available, low-cost, and accurate tool. Thus, screening and diagnosis of NAFLD can occur more rapidly due to these significant findings in clinical care.

## Supplementary Information

**Additional file 1: Supplementary Table 1.** Newcastle-Ottawa scale stars by domain.** Supplemental Figure 1.** Forest plot of Lipid accumulation product index mean difference grouped by continent in which study was conducted.** Supplemental Figure 2.** Forest plot of Lipid accumulation product index mean difference sub grouped by country in which study was conducted.** Supplemental Figure 3.** Forest plot of Lipid accumulation product index mean difference , sub grouped by diagnostic method of NAFLD.** Supplemental Figure 3.** Forest plot of Lipid accumulation product index mean difference, sub grouped by design of the study. CS: Cross-sectional.** Supplemental Figure 4. **Meta regression on Year of publication.** Supplemental Figure 5. **Meta-regression on female to male proportion.** Supplemental Figure 6. **Meta-regression on mean age of participants.

**Additional file 2.** Sensitivity Analysis.

## Data Availability

The datasets used for the analysis of this study are available as supplementary DATA.xlsx format files.

## Abbreviations

AUC: Area Under the Curve
DTA: Diagnostic Test Accuracy
HCC: Hepatocellular Carcinoma
IR: Insulin resistance
LAP: Lipid Accumulation Product
MD: Mean Difference
MetS: Metabolic Syndrome
MRI-PDFF: Magnetic Resonance Imaging Proton Density Fat Fraction
NAFLD: Non-Alcoholic Fatty Liver Disease
NASH: Nonalcoholic Steatohepatitis
NOS: Newcastle–Ottawa Scale
PRISMA: Preferred Reporting Items for Systematic Reviews and Meta-Analyses
PROSPERO: International Prospective Register of Systematic Reviews
SD: Standard Deviation
sROC: Summary Receiver Operating Characteristic
T2D: Type 2 Diabetes
TG: Triglyceride
WC: Waist Circumference
