# Supplementary material for: Lipid accumulation product (LAP) index for the diagnosis of nonalcoholic fatty liver disease (NAFLD): a systematic review and meta-analysis
Source: Lipids Health Dis. 2023 Mar 15;22:41. doi: 10.1186/s12944-023-01802-6 (PMC10015691; doi:10.1186/s12944-023-01802-6)
Supplement: Supplementary file 1 — Additional file 1: Supplementary Table 1. Newcastle-Ottawa scale stars by domain. Supplemental Figure 1. Forest plot of Lipid accumulation product index mean difference grouped by continent in which study was conducted. Supplemental Figure 2. Forest plot of Lipid accumulation product index mean difference sub grouped by country in which study was conducted. Supplemental Figure 3. Forest plot of Lipid accumulation product index mean difference , sub grouped by diagnostic method of NAFLD. Supplemental Figure 3. Forest plot of Lipid accumulation product index mean difference, sub grouped by design of the study. CS: Cross-sectional. Supplemental Figure 4. Meta regression on Year of publication. Supplemental Figure 5. Meta-regression on female to male proportion. Supplemental Figure 6. Meta-regression on mean age of participants. [file 12944_2023_1802_MOESM1_ESM.docx]

**Search Strategy**

**-Pubmed**

**("Lipid accumulation product" OR LAP) AND ("Non-alcoholic fatty liver disease" OR "Non-alcoholic Fatty Liver" OR "Non-alcoholic Fatty Livers" OR "Non-alcoholic Steatohepatitis" OR "Non-alcoholic Steatohepatitides" OR "Non-alcoholic hepatic steatosis" OR "Non-alcoholic Liver Steatosis" OR "Nonalcoholic fatty liver disease" OR "Nonalcoholic Fatty Liver" OR "Nonalcoholic Fatty Livers" OR "Nonalcoholic Steatohepatitis" OR "Nonalcoholic Steatohepatitides" OR "Nonalcoholic hepatic steatosis" OR "Nonalcoholic Liver Steatosis" OR "Non alcoholic fatty liver disease" OR "Non alcoholic Fatty Liver" OR "Non alcoholic Fatty Livers" OR "Non alcoholic Steatohepatitis" OR "Non alcoholic Steatohepatitides" OR "Non alcoholic hepatic steatosis" OR "Non alcoholic Liver Steatosis" OR NAFLD OR NASH)**

**-Scopus:**

**TITLE-ABS-KEY ("Lipid accumulation product" OR LAP) AND TITLE-ABS-KEY ("Non-alcoholic fatty liver disease" OR "Non-alcoholic Fatty Liver" OR "Non-alcoholic Fatty Livers" OR "Non-alcoholic Steatohepatitis" OR "Non-alcoholic Steatohepatitides" OR "Non-alcoholic hepatic steatosis" OR "Non-alcoholic Liver Steatosis" OR "Nonalcoholic fatty liver disease" OR "Nonalcoholic Fatty Liver" OR "Nonalcoholic Fatty Livers" OR "Nonalcoholic Steatohepatitis" OR "Nonalcoholic Steatohepatitides" OR "Nonalcoholic hepatic steatosis" OR "Nonalcoholic Liver Steatosis" OR "Non alcoholic fatty liver disease" OR "Non alcoholic Fatty Liver" OR "Non alcoholic Fatty Livers" OR "Non alcoholic Steatohepatitis" OR "Non alcoholic Steatohepatitides" OR "Non alcoholic hepatic steatosis" OR "Non alcoholic Liver Steatosis" OR NAFLD OR NASH)**

**-Web of Science:**

**#1: TI= ("Lipid accumulation product" OR LAP)**

**#2: AB= ("Lipid accumulation product" OR LAP)**

**#3: TI= ("Non-alcoholic fatty liver disease" OR "Non-alcoholic Fatty Liver" OR "Non-alcoholic Fatty Livers" OR "Non-alcoholic Steatohepatitis" OR "Nonalcoholic Steatohepatitides" OR "Non-alcoholic hepatic steatosis" OR "Non-alcoholic Liver Steatosis" OR "Nonalcoholic fatty liver disease" OR "Nonalcoholic Fatty Liver" OR "Nonalcoholic Fatty Livers" OR "Nonalcoholic Steatohepatitis" OR "Nonalcoholic Steatohepatitides" OR "Nonalcoholic hepatic steatosis" OR "Nonalcoholic Liver Steatosis" OR "Non alcoholic fatty liver disease" OR "Non alcoholic Fatty Liver" OR "Non alcoholic Fatty Livers" OR "Non alcoholic Steatohepatitis" OR "Non alcoholic Steatohepatitides" OR "Non alcoholic hepatic steatosis" OR "Non alcoholic Liver Steatosis" OR NAFLD OR NASH)**

**#4: AB= ("Non-alcoholic fatty liver disease" OR "Nonalcoholic Fatty Liver" OR "Non-alcoholic Fatty Livers" OR "Non-alcoholic Steatohepatitis" OR "Non-alcoholic Steatohepatitides" OR "Non-alcoholic hepatic steatosis" OR "Nonalcoholic Liver Steatosis" OR "Nonalcoholic fatty liver disease" OR "Nonalcoholic Fatty Liver" OR "Nonalcoholic Fatty Livers" OR "Nonalcoholic Steatohepatitis" OR "Nonalcoholic Steatohepatitides" OR "Nonalcoholic hepatic steatosis" OR "Nonalcoholic Liver Steatosis" OR "Non alcoholic fatty liver disease" OR "Non alcoholic Fatty Liver" OR "Non alcoholic Fatty Livers" OR "Non alcoholic Steatohepatitis" OR "Non alcoholic Steatohepatitides" OR "Non alcoholic hepatic steatosis" OR "Non alcoholic Liver Steatosis" OR NAFLD OR NASH)**

**#5: (#1 OR #2) AND (#3 OR #4)**

Supplementary Table 1. Newcastle-Ottawa scale stars by domain

| Author | Selection  (out of 4 stars) | Comparability  (out of 2 stars) | Exposure  (out of 3 stars) | Overall |
| --- | --- | --- | --- | --- |
| Ching-Lung Cheung | *** | ** | *** | 8 |
| DUAN Shaojie | ** | ** | ** | 6 |
| EDITH M. KOEHLER | *** | ** | ** | 7 |
| Evangeline Vassilatou | ** | ** | ** | 6 |
| Guotai Sheng | ** | ** | ** | 6 |
| Haijiang Dai | ** | ** | ** | 6 |
| I-Ting Lin | ** | ** | ** | 6 |
| Kartik Balankhe | ** | - | *** | 5 |
| Katarzyna Kozłowska-Petriczko | *** | ** | *** | 8 |
| Naiade Silveira Almeida | *** | - | *** | 6 |
| Raika Jamali | *** | ** | *** | 8 |
| Yan Zhang | ** | ** | ** | 6 |
| Yiting Liu | ** | ** | ** | 6 |
| Liuxin Zhang | *** | ** | ** | 7 |
| Xueyu Chen | *** | ** | ** | 7 |
| Moustafa Abd El Hamid Ali | ** | - | *** | 5 |

Sub group Analysis


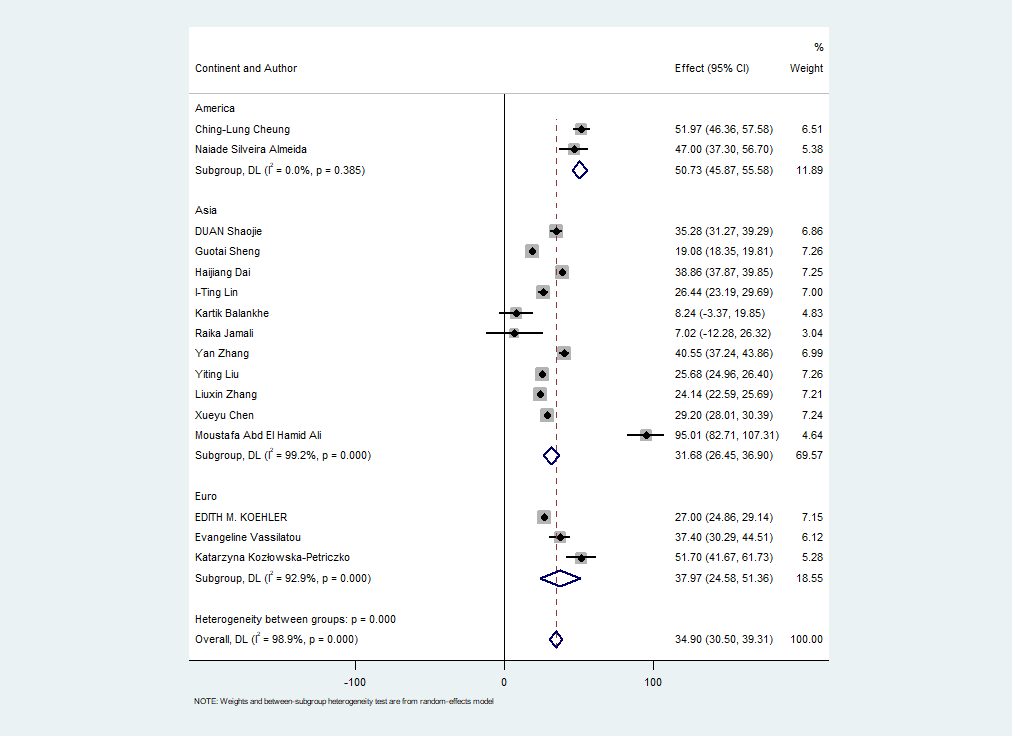


**Supplemental Figure 1:** Forest plot of Lipid accumulation product index mean difference grouped by continent in which study was conducted.


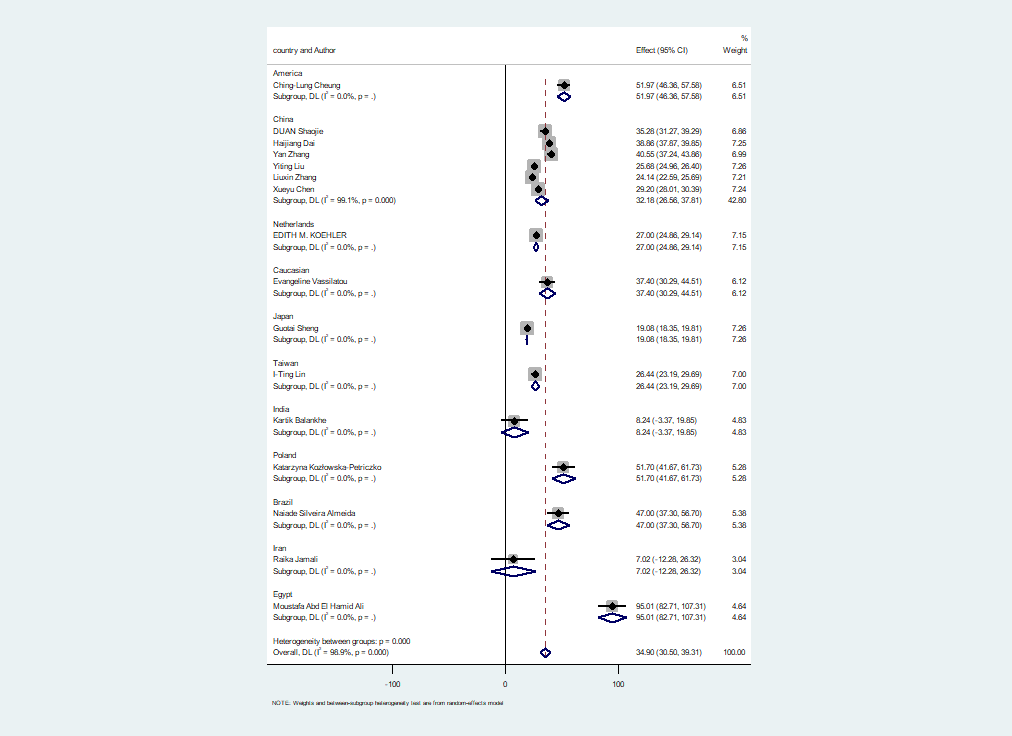


**Supplemental Figure 2:** Forest plot of Lipid accumulation product index mean difference sub grouped by country in which study was conducted.


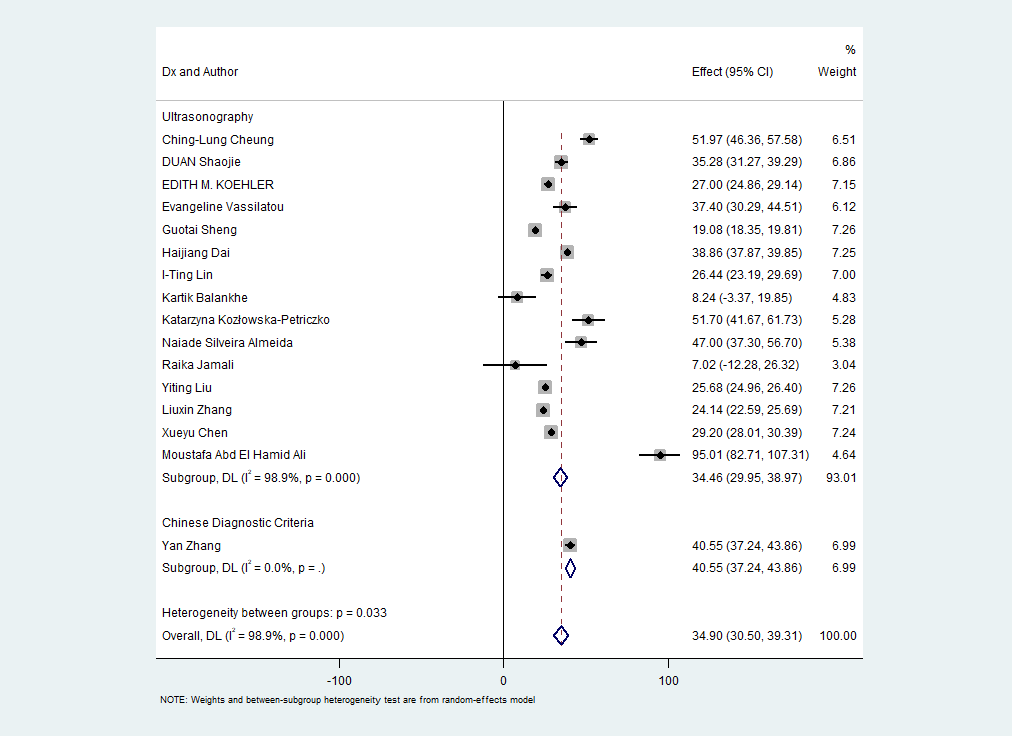


**Supplemental Figure 3:** Forest plot of Lipid accumulation product index mean difference , sub grouped by diagnostic method of NAFLD.


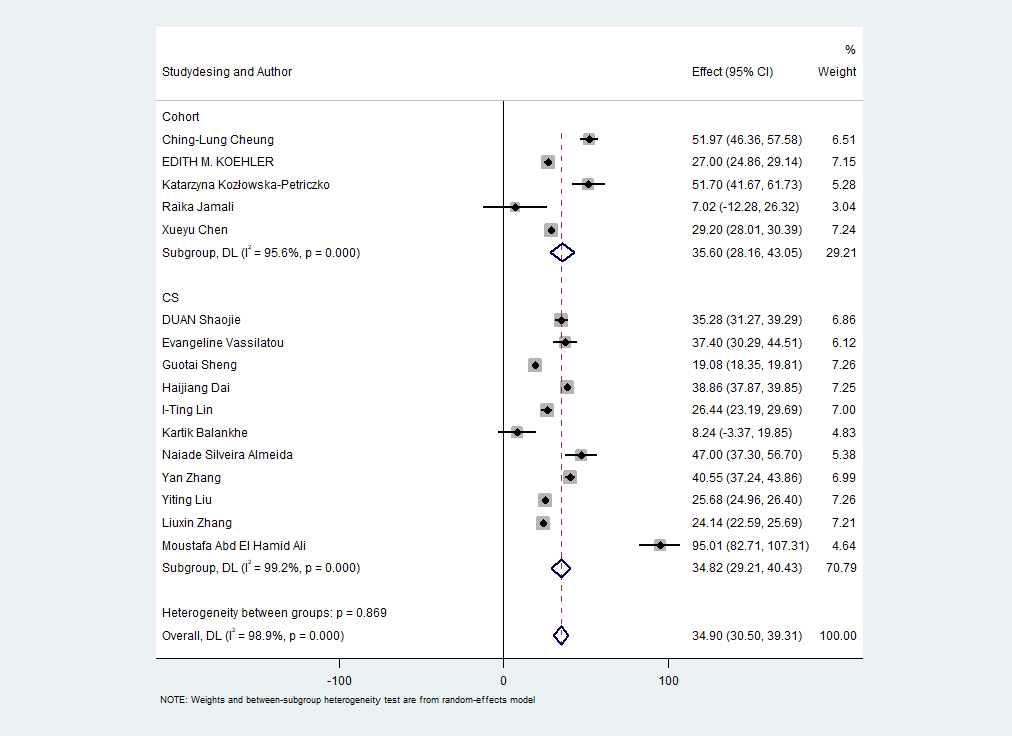


**Supplemental Figure 3:** Forest plot of Lipid accumulation product index mean difference, sub grouped by design of the study. CS: Cross-sectional.

Meta regression


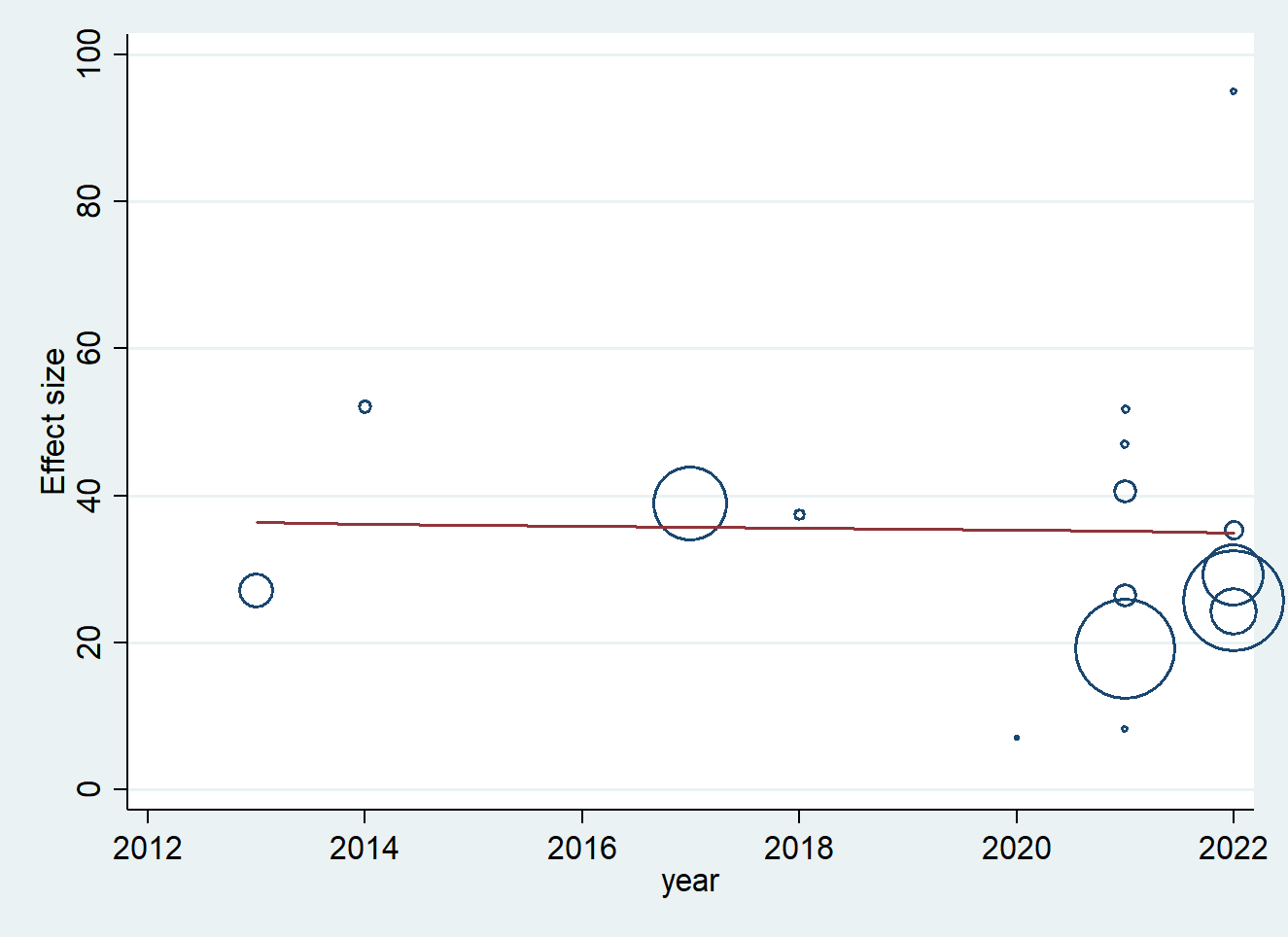


**Supplemental Figure 4** Meta regression on Year of publication

------------------------------------------------------------------------------

_ES | Coef. Std. Err. t P>|t| [95% Conf. Interval]

-------------+----------------------------------------------------------------

year | -.1479247 1.861987 -0.08 0.938 -4.141489 3.845639

_cons | 334.0941 3760.95 0.09 0.930 -7732.341 8400.529

------------------------------------------------------------------------------


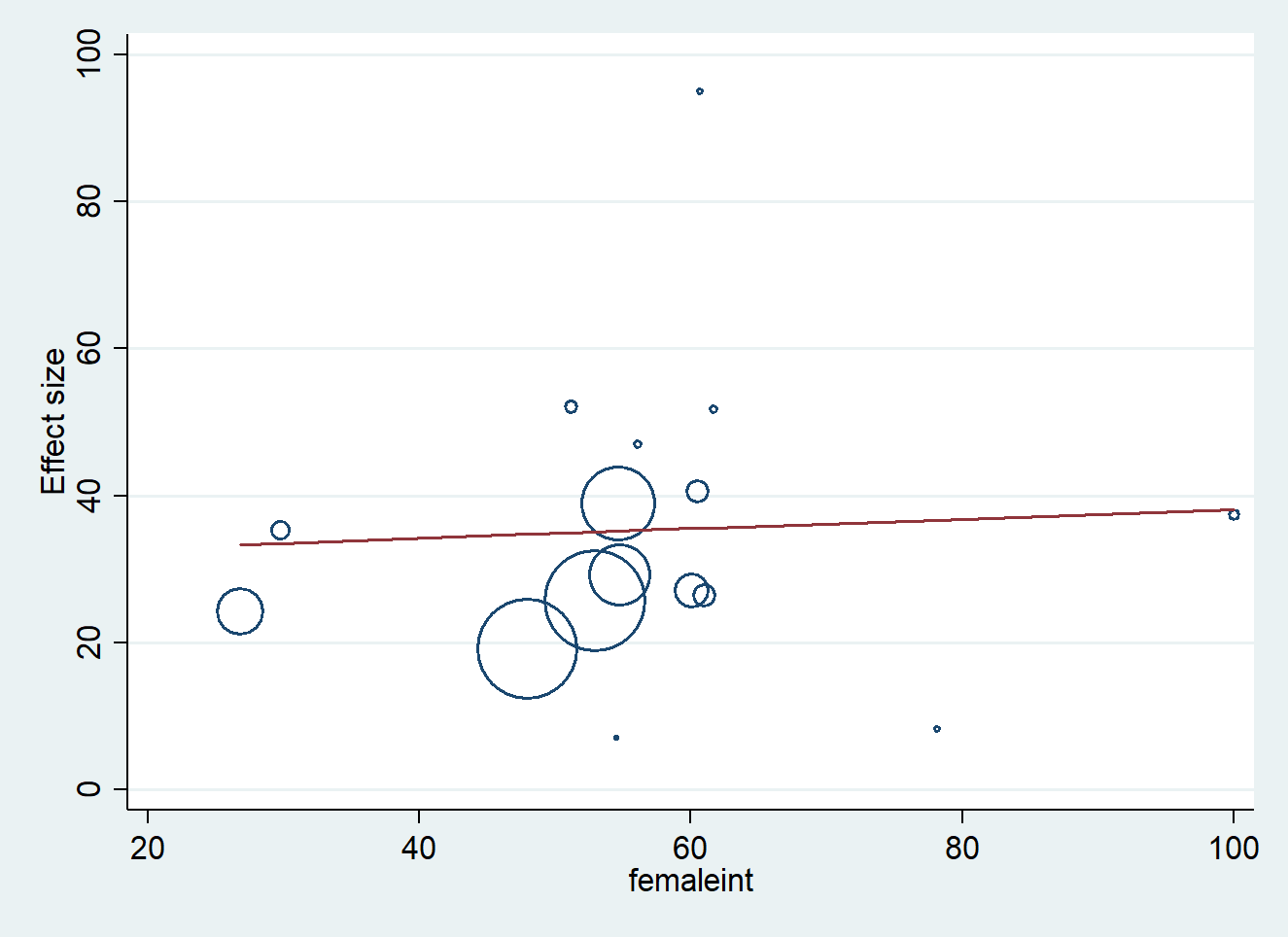


**Supplemental Figure 5** Meta-regression on female to male proportion

_ES | Coef. Std. Err. t P>|t| [95% Conf. Interval]

-------------+----------------------------------------------------------------

femaleint | .0655627 .3223559 0.20 0.842 -.6258221 .7569474

_cons | 31.58657 19.02669 1.66 0.119 -9.221633 72.39477


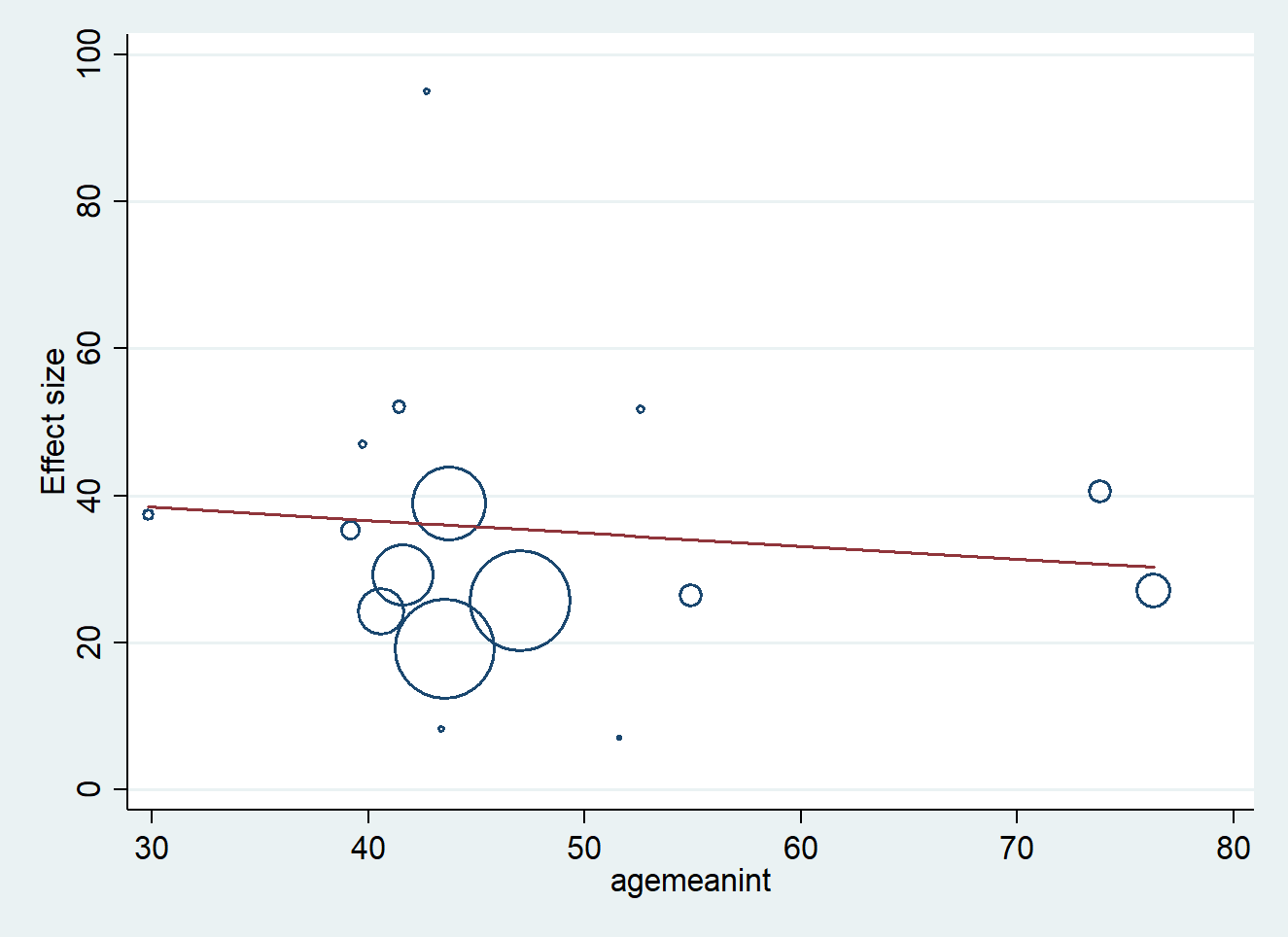


**Supplemental Figure 6** Meta-regression on mean age of participants

_ES | Coef. Std. Err. t P>|t| [95% Conf. Interval]

-------------+----------------------------------------------------------------

agemeanint | -.1762034 .4339502 -0.41 0.691 -1.106934 .7545272

_cons | 43.70445 21.32566 2.05 0.060 -2.034545 89.44344

------------------------------------------------------------------------------

Begg's test for small-study effects

Kendall's score = 6.00

SE of score = 22.211

z = 0.23

Prob > |z| = 0.8219

Egger's test for small-study effects: ------------------------------------------------------------------------------

Std_Eff | Coef. Std. Err. t P>|t| [95% Conf. Interval]

-------------+----------------------------------------------------------------

slope | 23.81494 2.632874 9.05 0.000 18.16799 29.46189

bias | 5.140409 3.29565 1.56 0.141 -1.928057 12.20888

Test of H0: no small-study effects P = 0.141
