# Supplementary figures and images for: Lipid accumulation product (LAP) index for the diagnosis of nonalcoholic fatty liver disease (NAFLD): a systematic review and meta-analysis
Source: Lipids Health Dis. 2023 Mar 15;22:41. doi: 10.1186/s12944-023-01802-6 (PMC10015691; doi:10.1186/s12944-023-01802-6)

Additional File 2. Sensitivity Analysis


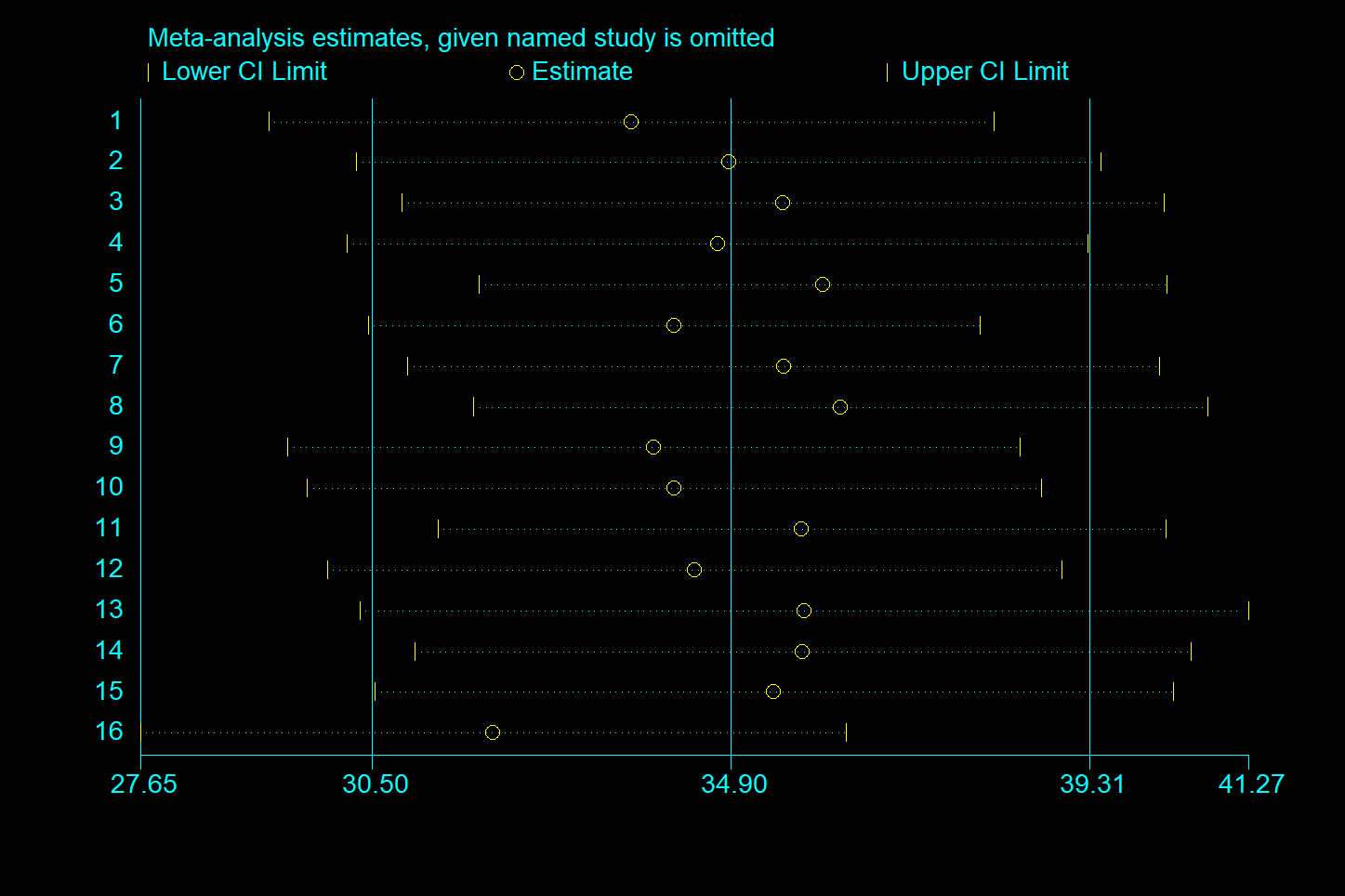

Supplement: Supplementary file 2 — Additional file 2. Sensitivity Analysis. [file 12944_2023_1802_MOESM2_ESM.docx]
